# Supplementary material for: Does access to finance condition firms’ green investment responses to environmental pressure? Evidence from Vietnam
Source: PLoS One. 2026 May 4;21(5):e0341960. doi: 10.1371/journal.pone.0341960 (PMC13138627; doi:10.1371/journal.pone.0341960)
Supplement: S1 Appendix — (DOCX) [file pone.0341960.s001.docx]

**Appendix Table A1. Robustness check using probit estimation**

| **Variables** | **(1) Probit** |
| --- | --- |
| Environmental pressure | 0.091* |
|  | (0.049) |
| Financial constraints | -0.156*** |
|  | (0.051) |
| Environmental pressure × Financial constraints | -0.072** |
|  | (0.035) |
| Firm size (log) | 0.184*** |
|  | (0.038) |
| Firm age | 0.003 |
|  | (0.002) |
| Exporter | 0.261*** |
|  | (0.065) |
| Foreign ownership | 0.058 |
|  | (0.056) |
| Industry fixed effects | Yes |
| Region fixed effects | Yes |
| Observations | 2,492 |
| Pseudo R² | 0.28 |

**Notes:** This table reports probit regression estimates as a robustness check. Robust standard errors are reported in parentheses. ***, **, and * denote statistical significance at the 1%, 5%, and 10% levels, respectively.
